# Glycated Albumin and Cardiovascular Mortality in CKD Stage V Patients with Diabetes Mellitus: A Five-Year Follow-Up Study

**DOI:** 10.3390/ijms27052215

**Published:** 2026-02-26

**Authors:** Ana Bulatovic, Nada Dimkovic, Svetlana Jelic, Aleksandar Jankovic, Tatjana Damjanovic, Verica Todorov-Sakic, Jelena Bjedov, Bojan Stopic, Petar Djuric, Radomir Naumovic

**Affiliations:** 1 Clinical Department for Nephrology, University Medical Center Zvezdara, 11000 Belgrade, Serbia; sashajan223@gmail.com (A.J.); damjanovictatjana1@gmail.com (T.D.); todorovverica@yahoo.com (V.T.-S.); jln_tosic@yahoo.com (J.B.); bojan.stopic@gmail.com (B.S.); djuricmed@gmail.com (P.D.); radomirnaumovic450@gmail.com (R.N.); 2 School of Medicine, University of Belgrade, 11000 Belgrade, Serbia; svetlana.jelic011@gmail.com; 3 Academy of Medical Sciences of the Serbian Medical Society, 11000 Belgrade, Serbia; dim@eunet.rs

**Keywords:** glycated albumin, glycemic markers, CKD Stage V, hemodialysis, diabetes mellitus, HbA1c, cardiovascular mortality, vrh obrasca

## Abstract

Glycemic assessment in patients with chronic kidney disease (CKD) Stage V on hemodialysis (HD) is limited by the inaccuracy of hemoglobin A1c (HbA1c), mainly due to anemia, shortened erythrocyte lifespan, and erythropoiesis-stimulating agent (ESA) therapy. Glycated albumin (GA), independent of erythrocyte turnover, may better reflect glycemic exposure. We evaluated the diagnostic performance and clinical utility of GA as a biomarker of poor glycemic control and cardiovascular risk in patients with diabetes mellitus (DM). A cross-sectional analysis and five-year prospective follow-up were conducted in three subgroups: HD+DM+ (*n* = 40), HD- DM+ (*n* = 15), and HD+DM– (*n* = 22). Glycemic markers (mean plasma glucose over 3 months (PG_3_m), GA, and HbA1c) were compared between groups. GA levels were significantly higher in HD+DM+ patients (*p* < 0.001) and showed the strongest correlation with PG_3_m. GA independently predicted poor glycemic control (OR 3.23; 95% CI 1.54–6.77; *p* = 0.002) and demonstrated high diagnostic accuracy (AUC 0.873; optimal cut-off 10%). During the five-year follow-up, CV mortality was 35%, and 85% of deceased patients had GA > 10%. Although Cox regression did not reach statistical significance, GA showed a consistent trend toward higher CV mortality risk after adjustment for age and HD duration (HR 2.56; 95% CI 0.57–11.44; *p* = 0.21), whereas HbA1c was not prognostic (HR 1.39; 95% CI 0.49–3.91; *p* = 0.28). GA appears to be a clinically useful marker of glycemic control in diabetic patients with CKD Stage V receiving maintenance hemodialysis. In this cohort, GA showed high diagnostic accuracy and a more balanced sensitivity/specificity profile compared with HbA1c, with a consistent trend toward association with cardiovascular mortality. In this regard, the wider use of this marker in clinical practice is worth considering. Nevertheless, larger prospective studies are warranted to validate these observations.

## 1. Introduction

Diabetes mellitus (DM) is the leading global driver of chronic kidney disease (CKD) progression and remains the primary cause of CKD Stage V worldwide [1,2]. CKD Stage V represents end-Stage kidney disease characterized by a severely reduced glomerular filtration rate (eGFR < 15 mL/min/1.73 m^2^) and typically requires renal replacement therapy. Patients with DM and CKD Stage V carry an exceptionally high cardiovascular risk, with mortality rates several-fold higher than in age-matched individuals without kidney disease [3]. In this context, accurate assessment of glycemic control becomes clinically critical and prognostically decisive, as chronic hyperglycemia contributes to accelerated atherosclerosis, systemic inflammation, endothelial dysfunction, and myocardial remodeling.

Despite its widespread use, glycated hemoglobin (HbA1c) performs poorly as a measure of glycemic control in advanced CKD and patients with diabetes receive maintenance hemodialysis. Its validity is compromised by shortened erythrocyte lifespan, hemolysis, frequent transfusions, iron deficiency, treatment with erythropoiesis-stimulating agents (ESAs), and profound alterations in red blood cell turnover characteristic of uremia [4,5,6]. As a result, HbA1c consistently underestimates true glycemic exposure, potentially masking clinically relevant variability and leading to suboptimal therapeutic decisions in the hemodialysis setting.

Glycated albumin (GA) has emerged as a biologically stronger and clinically more reliable alternative in this population. Unlike HbA1c, GA is independent of erythrocyte kinetics and reflects glycemia over a shorter interval (2–3 weeks), offering improved sensitivity to fluctuations in blood glucose and better correlation with actual glycemic patterns in CKD Stage V [7,8,9]. GA also represents a major circulating Amadori product and may contribute directly to vascular injury through oxidative stress, inflammation, foam cell formation, and acceleration of coronary atherosclerosis—pathways highly relevant in hemodialysis patients with markedly increased cardiovascular mortality. Although hypoalbuminemia may occur in this setting, GA remains a useful and reliable glycemic marker, especially when interpreted in the context of serum albumin levels [10,11,12].

Although the clinical utility of GA in diabetic kidney disease has become increasingly recognized, key knowledge gaps persist. Given the established associations between GA and vascular inflammation, endothelial dysfunction, and atherosclerotic progression, its prognostic role in cardiovascular mortality among hemodialysis patients warrants clarification. However, its prognostic value in CKD Stage V patients on chronic hemodialysis, including its capacity to predict inadequate glycemic control and long-term cardiovascular outcomes, remains insufficiently defined. Existing studies, although increasingly longitudinal, remain limited by heterogeneous CKD populations, varying dialysis exposure, short follow-up periods, or insufficient dialysis-specific analyses [10,13,14,15]. Given these gaps, a rigorous evaluation of GA as a clinically meaningful biomarker in diabetic maintenance hemodialysis patients is needed.

In our previous investigations of glycated albumin in advanced CKD and dialysis populations, we observed that GA may provide complementary metabolic and potential prognostic information beyond traditional glycemic markers. Building upon this line of research, the present study was designed to provide a comprehensive longitudinal evaluation of the association between GA and cardiovascular mortality over a five-year follow-up period in a well-characterized CKD Stage V cohort undergoing maintenance hemodialysis [16].

Therefore, the aim of this study was to assess the clinical utility of glycated albumin in diabetic patients with CKD Stage V on chronic hemodialysis, to determine its predictive accuracy for poor glycemic control, and to evaluate its independent association with cardiovascular mortality over a five-year follow-up period.

## 2. Results

### 2.1. Baseline Clinical Characteristics

Baseline clinical characteristics included cardiovascular events (CVEs) and hypertension (HTA), both established determinants of cardiovascular risk in patients with advanced CKD. Namely, CVEs and HTA with prolonged CKD can lead to serious complications such as heart failure and even sudden cardiac death. There were no significant differences in age or sex distribution across the three groups (Table 1). BMI differed significantly between groups, with lower values observed in the HD+DM+ cohort compared with the HD−DM+ group. Previous CVEs, including myocardial infarction and ischemic stroke, were more frequent in the HD+DM+ group.

The HD+DM+ group had significantly lower serum albumin and hemoglobin levels compared with the other cohorts (Table 2). No statistically significant differences in lipid parameters were observed across the groups.

### 2.2. Parameters of Glycemic Control

All glycemic markers were highest among diabetic hemodialysis patients, confirming that dialysis contributes to greater metabolic instability and glycemic variability. The significantly elevated GA levels in HD+DM+ patients compared with diabetic individuals without CKD highlight the superior sensitivity of GA to real glycemic exposure under conditions of altered erythrocyte kinetics (Table 3).

Spearman correlation analysis was performed to evaluate the relationships between PG_3_m, HbA1c, and GA in diabetic subgroups. In the HD+DM+ group, all glycemic markers showed strong and statistically significant positive correlations, with the strongest association observed between PG_3_m and HbA1c (ρ = 0.768, *p* < 0.001). In the HD–DM+ group, correlations were generally weaker, and the association between PG_3_m and GA did not reach statistical significance (ρ = 0.323, *p* = 0.240). Detailed results are presented in Table 4.

In univariate logistic regression analyses, all glycemic markers (PG, PG_3_m, HbA1c, and GA) were significantly associated with poor glycemic control in the HD+DM+ group (Table 5). In the multivariate model adjusted for serum hemoglobin and albumin, PG_3_m (per 1 mmol/L: OR 3.96; 95% CI 1.06–14.78; *p* = 0.041) and GA (per 1%: OR 3.23; 95% CI 1.54–6.77; *p* = 0.002) remained independent predictors. These findings suggest that GA provides clinically relevant diagnostic information for identifying inadequate glycemic control in patients with diabetes receiving maintenance hemodialysis, even after accounting for key hematologic and nutritional parameters.

**Figure 1:**
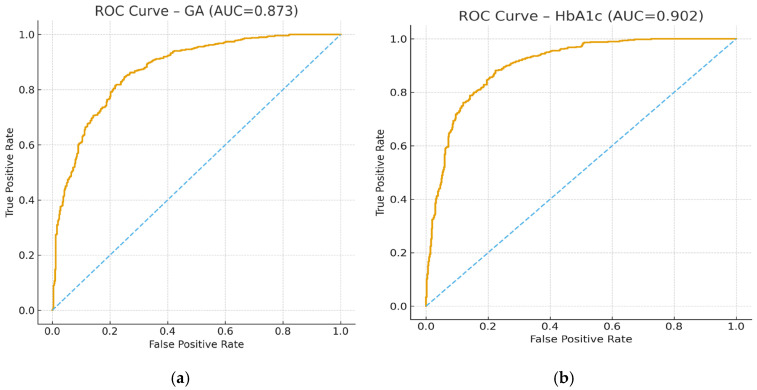
(**a**) ROC Curve—Glycated Albumin, (**b**) ROC Curve—HbA1c. AUC-Area Under the Curve. The yellow solid line represents the ROC curve for GA and HbA1c. The blue dashed line indicates the reference line of no discrimination (AUC = 0.5).

Receiver operating characteristic (ROC) curve for glycated albumin (GA) in identifying poor glycemic control defined by elevated PG_3_m in HD+DM+ patients. AUC = 0.873 (SE 0.04; 95% CI 0.79–0.94; *p* < 0.001). The optimal cut-off value was 10% (Youden index), yielding a sensitivity of 80% and a specificity of 78%. The diagonal line represents the reference line of no discrimination (Figure 1a).

Receiver operating characteristic (ROC) curve for HbA1c in identifying poor glycemic control defined by elevated PG_3_m in HD+DM+ patients. AUC = 0.902 (SE 0.03; 95% CI 0.83–0.96; *p* < 0.001). The optimal cut-off value was 6.5% (Youden index), with sensitivity of 64% and specificity of 100%. The diagonal line represents the reference line of no discrimination (Figure 1b).

Both GA and HbA1c demonstrated high diagnostic accuracy; however, GA provided a more favorable balance between sensitivity and specificity at a clinically practical cut-off of 10%. In contrast, HbA1c achieved maximal specificity at the expense of substantially reduced sensitivity, which may limit its clinical interpretability in CKD Stage V characterized by anemia, ESA therapy, and altered erythrocyte turnover. Collectively, these findings support GA as a clinically useful biomarker for identifying inadequate glycemic control in diabetic hemodialysis patients.

### 2.3. Predictors of Cardiovascular Outcome

Despite its high AUC for glycemic control, HbA1c did not translate into prognostic value for cardiovascular mortality, likely due to erythrocyte turnover–related distortions that are independent of cardiovascular pathophysiology. Clinical and laboratory parameters according to GA and HbA1c categories are presented in Table 6.

During the 5-year follow-up period, cumulative mortality in hemodialysis diabetic patients (in total 40 patients) was 65% (26 patients) and cardiovascular mortality 35% (14 patients). Among all HD+DM+ patients who died during follow-up (n = 26), 85% had GA > 10%. 

There were no significant differences in inflammatory, nutritional, or hematologic parameters between groups stratified by GA or HbA1c thresholds, indicating that baseline comorbidity-related characteristics were largely similar across glycation categories. Slightly higher CRP levels and ESA doses were observed in patients with elevated glycation markers, suggesting a potential contribution of inflammation and treatment intensity to poorer glycemic control, although these trends did not reach statistical significance.

**Figure 2:**
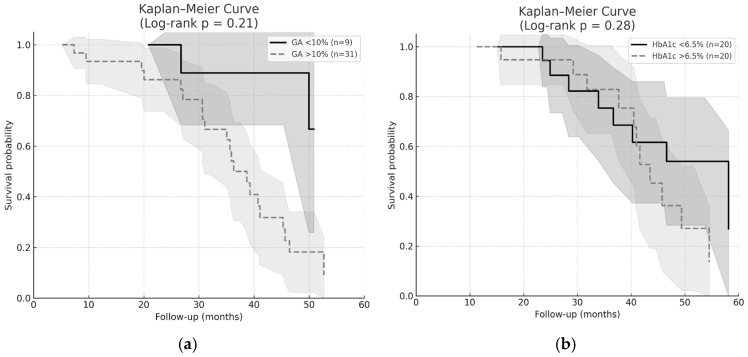
(**a**) Kaplan–Meier Curve—GA, (**b**) Kaplan–Meier Curve—HbA1c. The grey areas represent the 95% confidence intervals (95% CI).

Kaplan–Meier survival analysis demonstrated visual separation of cardiovascular mortality curves between GA strata (GA < 10% vs. GA > 10%), indicating a trend toward higher cardiovascular risk among patients with elevated GA (Figure 2a). However, the difference did not reach statistical significance (log-rank *p* = 0.21).

In multivariate Cox regression models adjusted for age and HD duration, patients with GA > 10% exhibited a 2.56-fold higher risk of cardiovascular mortality compared with those with GA < 10% (HR 2.56, 95% CI 0.57–11.44; *p* = 0.21). Although statistical significance was not achieved, the direction and magnitude of the hazard ratio suggest a potentially stronger prognostic signal for GA.

In contrast, HbA1c-based stratification (>6.5% vs. <6.5%) did not result in meaningful survival discrimination (Figure 2b). The adjusted hazard ratio for HbA1c > 6.5% was 1.39 (95% CI 0.49–3.91; *p* = 0.28), indicating a weaker association with cardiovascular mortality compared with GA. Kaplan–Meier survival curves stratified by GA and HbA1c are presented in Figure 2a and Figure 2b, respectively. Shaded areas represent 95% confidence intervals.

## 3. Discussion

In this study, we found that glycated albumin (GA) provides a more informative assessment of glycemic exposure than HbA1c in patients with CKD Stage V receiving chronic hemodialysis and may be more closely related to cardiovascular risk during long-term follow-up. Importantly, this study extends prior evidence where we have already mentioned the advantage of determining GA over traditional markers of glycemic control. With this work, we supported our initial findings with more relevant longitudinal CV outcome data [16]. These findings are consistent with the well-established limitations of HbA1c in advanced CKD, where shortened erythrocyte life span, hemodilution, erythropoiesis-stimulating agent therapy, and chronic inflammation contribute to systematic underestimation of glycemic exposure [4,5,6]. Because GA formation is independent of erythrocyte turnover, it reflects glycemic exposure over the preceding 2–3 weeks and remains relatively stable despite metabolic disturbances in the uremic milieu. Moreover, GA has been reported to be more reliable than HbA1c in advanced CKD and hemodialysis settings, largely because it is not affected by shortened erythrocyte survival; additionally, higher GA levels have been associated with coronary disease and increased cardiovascular risk [7,8,9]. This likely explains the greater clinical interpretability of GA in our cohort. Furthermore, GA demonstrated stronger correlations with mean plasma glucose and higher diagnostic accuracy for identifying inadequate glycemic control compared with HbA1c. Higher GA values were associated with higher cardiovascular mortality, whereas HbA1c showed no prognostic value. Although causality cannot be inferred and Cox regression did not reach statistical significance, the overall pattern of findings suggests that GA may better capture clinically relevant glycemic exposure and cardiovascular vulnerability in this high-risk population.

In the present study, GA demonstrated a stronger correlation with PG_3_m and remained an independent predictor of poor glycemic control (OR 3.23), findings consistent with Peacock et al. [7] and Lee et al. [8], who previously established GA as a superior glycemic marker in advanced CKD and HD populations. These findings are also consistent with our earlier investigations in dialysis populations, which suggested that GA may offer clinically relevant information beyond HbA1c in advanced CKD settings. More recent studies reinforce this concept: Meyer et al. [17] further supported the clinical utility of GA for glycemic assessment in hemodialysis patients, particularly compared with HbA1c, whereas Shafi et al. [18] reported that glycated albumin and fructosamine were associated with mortality and adverse clinical outcomes in hemodialysis patients, supporting the prognostic relevance of non–HbA1c glycemic markers in this population.

Our findings suggest that GA may represent a clinically relevant biomarker for glycemic assessment and cardiovascular risk stratification in patients with diabetes receiving maintenance hemodialysis. The potential clinical utility of GA was further supported by the observation that 85% of patients who died during follow-up had GA > 10%, indicating that this threshold may help identify individuals at particularly high risk; however, this finding should be interpreted cautiously given the limited cohort size. Although Cox regression models did not achieve statistical significance—likely due to limited sample size and the limited number of events—the direction and magnitude of hazard ratios (HR > 2.5), despite wide confidence intervals, pointed toward a potentially clinically meaningful association and were in line with larger studies reporting GA as an independent prognostic marker in advanced CKD populations, including patients receiving dialysis CKD [13,14,15]. Importantly, our study additionally provides absolute outcome estimates, with 65% overall mortality and 35% cardiovascular mortality over 5 years, which may strengthen the clinical interpretation of prior evidence regarding the prognostic value of GA in advanced CKD/HD populations.

Importantly, the prognostic relevance of glycated albumin has also been demonstrated outside the dialysis population. In a large community-based cohort, Selvin et al. showed that elevated GA levels were independently associated with increased cardiovascular and all-cause mortality, supporting the biological plausibility of GA as a robust risk marker across different clinical settings [19]. In line with these observations, Shen Y et al. [9] demonstrated that elevated GA predicts progression of coronary atherosclerosis, and Fukuoka et al. [15] reported that higher GA levels correlate with increased long-term mortality in diabetic dialysis patients.

Kaplan–Meier survival curves in our study showed clear separation between GA <10% and GA > 10% groups, suggesting a trend toward higher cardiovascular risk among patients with elevated GA. This finding is consistent with prior dialysis-based evidence indicating that GA may provide prognostic information beyond HbA1c, including prediction of cardiovascular hospitalization and length of hospital stay in diabetic patients on dialysis [14]. In contrast, HbA1c failed to discriminate risk in our cohort, aligning with previous reports demonstrating its limited biological validity in CKD Stage V due to altered erythrocyte kinetics, anemia, and ESA therapy [4,5,6].

Baseline differences across the study groups were clinically consistent with the hemodialysis setting. Compared with the other cohorts, the HD+DM+ group demonstrated lower serum albumin and hemoglobin levels, reflecting the combined burden of chronic inflammation, nutritional vulnerability, and impaired erythropoiesis commonly observed in end-Stage kidney disease. The lower BMI in this group further supports the presence of uremia-related catabolism and metabolic stress typical for advanced CKD. Notably, no significant between-group differences were observed in lipid parameters, which may be explained by the fact that uremia can attenuate conventional dyslipidemia patterns and lead to a convergence of lipid profiles irrespective of diabetes status.

Notably, GA was not significantly correlated with CRP, serum albumin, or ESA dosage in our cohort, suggesting relative robustness of GA against variability in inflammatory and nutritional parameters in this dialysis population. This stability of GA, even in the context of inflammatory variability, has been previously emphasized by Koga and Kasayama [11].

Collectively, these findings support the growing consensus that GA is a more robust and clinically meaningful marker of glycemic regulation—and potentially a more informative indicator of cardiovascular risk—compared with HbA1c in diabetic patients undergoing hemodialysis. Given the exceptionally high cardiovascular burden in this population, integrating GA into routine assessment may support risk-oriented follow-up, enhance therapeutic individualization, and facilitate early identification of high-risk individuals, pending confirmation in larger multicenter prospective studies.

### 3.1. Clinical Implications

Clinically, GA may provide a more reliable measure of glycemic control in hemodialysis patients, particularly in those with anemia, ESA therapy, or altered erythrocyte lifespan, where HbA1c is known to be misleading. This is especially relevant in patients receiving high doses of erythropoiesis-stimulating agents (ESAs) or in individuals whose HbA1c values appear discordant with observed glycemic variability. In such scenarios, GA measurement may offer additional metabolic insight and help prevent misclassification of glycemic control when HbA1c underestimates true glycemic exposure.

Furthermore, GA-based stratification may assist clinicians in identifying patients at increased cardiovascular risk who could benefit from closer metabolic surveillance and tailored therapeutic adjustment. A GA threshold >10% may serve as a pragmatic early warning indicator prompting intensified monitoring within the hemodialysis setting.

Given the exceptionally high cardiovascular burden in diabetic HD patients, incorporation of GA into routine metabolic assessment may support more individualized, risk-oriented follow-up strategies. Nevertheless, prospective multicenter studies are required to validate GA-guided management approaches and to confirm its prognostic role in this population.

### 3.2. Limitations

Several limitations of this study should be acknowledged. The single-center design and relatively small sample size, particularly in the prospective cohort, limited the statistical power of survival analyses and precluded causal inference. In addition, therapeutic strategies were not guided by GA levels, preventing assessment of GA-directed interventions. Furthermore, broader standardization of GA assays and greater accumulation of clinical experience are needed before widespread clinical implementation can be considered. Therefore, these findings should be interpreted as exploratory. Larger, prospective, multicenter studies are warranted to validate our results and to clarify the clinical and prognostic role of GA in patients with CKD Stage V undergoing hemodialysis.

## 4. Materials and Methods

This study used a combined analytical cross-sectional and prospective cohort design. The cross-sectional component compared biochemical and clinical parameters across three predefined groups: (1) HD+DM+ (n = 40), chronic hemodialysis patients with type 2 diabetes mellitus; (2) HD–DM+ (n = 15), patients with type 2 diabetes mellitus without chronic kidney disease (eGFR ≥ 60 mL/min/1.73 m^2^ and no evidence of albuminuria), not requiring dialysis; and (3) HD+DM– (n = 22), hemodialysis patients without diabetes mellitus. The prospective component followed the HD+DM+ cohort for five years to evaluate glycated albumin (GA) as a predictor of cardiovascular mortality.

Hemodialysis procedures followed a standardized protocol consisting of three weekly sessions lasting 4–6 h, using high-flux polysulfone dialyzers (FX80, Fresenius Medical Care, Bad Homburg, Germany, 1.8 m^2^ surface), blood flow rates of 250–320 mL/min, dialysate flow rates of 500–700 mL/min, and vascular access via arteriovenous fistula or tunneled catheter. Ultrafiltration was individualized, and dialysis adequacy was maintained at a weekly Kt/V ≥ 1.2. Erythropoiesis-stimulating agent therapy and intravenous iron supplementation were administered according to institutional guidelines.

Type 2 diabetes mellitus was confirmed by documented diagnosis or according to American Diabetes Association criteria (fasting plasma glucose ≥ 7.0 mmol/L, HbA1c ≥ 6.5%, or a positive oral glucose tolerance test). Diabetic patients were required to have stable antidiabetic therapy for at least four weeks prior to enrollment. Exclusion criteria included active malignancy, sepsis, acute diabetic crises, severe electrolyte disturbances, advanced liver disease, nephrotic syndrome or severe hypoalbuminemia (serum albumin < 30 g/L) of non-uremic etiology, recent blood transfusion (<1 month), and inability to provide informed consent.

Glycemic markers included 3-month mean plasma glucose (PG_3_m), glycated albumin (GA), and HbA1c, which are widely used glycemic indices in dialysis populations [6,7]. PG_3_m was calculated as the arithmetic mean of three plasma glucose measurements obtained during the 3-month observation period. The GA and HbA1c thresholds used to define poor glycemic control (GA > 10% and HbA1c > 6.5%) were derived from ROC curve analyses by selecting the optimal cut-off values for predicting elevated PG_3_m. Thus, these biomarker-based categories were data-driven and anchored to PG_3_m as the reference measure of glycemic exposure in this cohort. Poor glycemic control was predefined as GA > 10% or HbA1c > 6.5%; these thresholds are consistent with cut-offs and classification approaches reported in previous studies [6,7].

All laboratory samples were collected after overnight fasting. Although the study included a five-year clinical follow-up, glycated albumin (GA) and HbA1c were measured at baseline and analyzed immediately after sample processing. No long-term storage of samples was performed. For GA determination, blood was collected into serum tubes without anticoagulant. After clotting at room temperature, samples were centrifuged at 3000 rpm for 10 min at room temperature within one hour of collection. Serum was immediately analyzed using a commercially available ELISA kit (EIAab, Wuhan EIAab Science Co., Ltd., Wuhan, China).

HbA1c was measured from whole blood collected in EDTA-containing tubes using an immunochemical assay based on monoclonal antibody detection on an automated biochemical analyzer (Olympus AU 400, Olympus Diagnostics, Tokyo, Japan). The method is standardized according to International Federation of Clinical Chemistry (IFCC) reference measurement procedures and aligned with National Glycohemoglobin Standardization Program (NGSP)/Diabetes Control and Complications Trial (DCCT) reference values. Results were expressed in percentage (%) units and IFCC units (mmol/mol). Analytical performance characteristics were in accordance with manufacturer specifications. The assay demonstrated acceptable intra-assay and inter-assay precision, with coefficients of variation below 3%. All samples were analyzed immediately after collection without long-term storage. Although analytically robust, the clinical interpretation of HbA1c in patients with CKD Stage V may be influenced by altered erythrocyte turnover, anemia, and erythropoiesis-stimulating agent therapy.

Plasma glucose was measured using the hexokinase method; serum albumin by the bromocresol green method; lipid profile by enzymatic assays; and C-reactive protein by high-sensitivity immunoturbidimetry. In hemodialysis patients, blood samples were obtained prior to the mid-week dialysis session.

In the prospective HD+DM+ cohort, the primary end point was cardiovascular mortality. Secondary endpoints included all-cause mortality and cardiovascular hospitalization. Censoring occurred at kidney transplantation, transfer to another dialysis center, dialysis withdrawal, or survival to the end of the study period. Mortality outcomes were verified using hospital medical records and the National Health Registry.

### Statistical Analysis

Data distribution was assessed using the Shapiro–Wilk test. Normally distributed variables were presented as mean ± standard deviation (SD), whereas non-normal variables were expressed as median with interquartile range (IQR). Group comparisons were performed using the independent-samples *t*-test for normally distributed two-group comparisons, the Mann–Whitney U test for non-normally distributed two-group comparisons, one-way ANOVA with LSD post hoc analysis for three-group comparisons, and the Kruskal–Wallis test for non-normal multi-group comparisons. Categorical variables were compared using the χ^2^ test or Fisher’s exact test, as appropriate.

Correlations between glycemic markers (PG_3_m, GA, and HbA1c) were assessed using Spearman’s rank correlation coefficient (ρ). Univariate and multivariate logistic regression analyses were performed to identify predictors of poor glycemic control. Variables with *p* < 0.10 in univariate analyses were included in the multivariate model. Model assumptions, including multicollinearity, were evaluated prior to final adjustment. Receiver operating characteristic (ROC) curve analysis was used to assess diagnostic performance, with calculation of the area under the curve (AUC), sensitivity, and specificity. Survival analysis was performed using Kaplan–Meier curves with log-rank testing, and Cox proportional hazards regression was applied to estimate hazard ratios for cardiovascular mortality, adjusted for relevant clinical covariates.

A two-tailed *p*-value < 0.05 was considered statistically significant. Statistical analyses were performed using SPSS version 26.0 (IBM Corp., Armonk, NY, USA).

The study was conducted in accordance with the Declaration of Helsinki and approved by the Ethics Committee of Zvezdara University Medical Center (No. 05-09/2025; 2 September 2025).

## 5. Conclusions

Glycated albumin (GA) appears to be a more accurate and clinically informative biomarker of glycemic control than HbA1c in diabetic patients undergoing hemodialysis. In our cohort, GA more closely reflected clinically relevant glycemic exposure, remained independently associated with poor glycemic regulation, and showed a consistent trend toward an association with cardiovascular mortality despite limited statistical power. These observations support the concept that GA, in contrast to HbA1c, is less affected by CKD Stage V–related alterations in erythrocyte kinetics and may therefore provide complementary diagnostic and potential prognostic information. Incorporation of GA into routine clinical assessment may offer a useful adjunct for identifying high-risk individuals and supporting cardiovascular risk stratification in this vulnerable population. In routine dialysis practice, GA assessment may be particularly valuable in patients with discordant HbA1c values or unexplained cardiovascular vulnerability. Further large-scale, multicenter longitudinal studies are warranted to confirm these findings and to define clinically meaningful GA-based thresholds.

## Figures and Tables

**Table 1:** Baseline demographic and clinical characteristics of the study groups.

Variable	I Group HD+DM+ *n*= 40	II Group HD–DM+ *n* = 15	III Group HD+DM– *n* = 22	*p*-Value
Age (years), mean ± SD	61.2 ± 12.8	59.2 ± 12.1	62.0 ± 11.6	0.787
Gender (women/men, %)	35.0/65.0	66.7/33.3	45.5/54.5	0.108
BMI (kg/m^2^), mean ± SD	24.2 ± 5.4	29.4 ± 7.0	23.2 ± 3.3	0.002 *
Waist circumference (cm), mean ± SD	93.5 ± 15.3	101.6 ± 16.3	90.3 ± 11.4	0.068
Previous CVE (%)	85.0	46.7	74.5	0.015 **
History of HTA (%)	87.5	80.0	86.4	0.774
History of smoking (%)	25.0	33.3	43.0	0.215
DM duration (years), median (IQR)	15 (3)	8 (8)	—	0.02 †
HD duration (years), median (IQR)	2 (6)	—	3.5 (7)	0.169 ‡

ANOVA (LSD post hoc): * χ^2^ test: I vs. II *p* = 0.002; II vs. III *p* = 0.001. ** χ^2^ test: I vs. II *p* = 0.004. † Comparison only between diabetes groups (I vs. II). ‡ Comparison only between hemodialysis groups (I vs. III). Data are presented as mean ± SD unless indicated otherwise (median [IQR] for non-normal distributions). All diabetic participants had type 2 diabetes mellitus (T2DM). CVE—cardiovascular events; HTA—hypertension; HD—hemodialysis; DM—diabetes mellitus.

**Table 2:** Clinical and laboratory characteristics of the three study groups.

Variable	I Group HD+DM+ *n* = 40	II Group HD–DM+ *n* = 15	III Group HD+DM– *n* = 22	*p*-Value
Serum albumin (g/L)	35.3 ± 6.2	41.9 ± 9.5	37.2 ± 2.8	0.004 *
Total cholesterol (mmol/L)	4.6 ± 1.4	4.8 ± 1.2	4.7 ± 0.9	0.797
LDL cholesterol (mmol/L)	2.4 ± 0.7	2.6 ± 0.8	2.5 ± 0.7	0.694
HDL cholesterol (mmol/L)	1.0 ± 0.4	0.9 ± 0.1	1.1 ± 0.3	0.262
Triglycerides (mmol/L)	2.3 ± 2.3	2.5 ± 1.8	2.2 ± 1.0	0.876
Hemoglobin (g/dL)	10.0 ± 0.8	12.7 ± 1.1	10.0 ± 0.8	<0.001 **
Hematocrit (%)	32.9 ± 4.0	38.4 ± 3.5	34.5 ± 3.2	<0.001 ***
Ferritin (µg/L)	323.9 ± 236.3	157.2 ± 194.4	300.5 ± 192.3	0.042 #
Weekly ESA dose (IU) †	6328 ± 5027	—	4925 ± 3403	0.272 ‡
Kt/V †	1.2 ± 0.3	—	1.3 ± 0.3	0.590 ‡
CRP (mg/L), median (IQR)	23.9 (70.6)	12.4 (26.8)	6.7 (5.6)	0.444

* ANOVA (LSD post hoc): I vs. II *p* = 0.01; II vs. III *p* = 0.02. ** ANOVA (LSD post hoc): I vs. II *p* < 0.001; II vs. III *p* < 0.001. *** ANOVA (LSD post hoc): I vs. II *p* < 0.001; II vs. III *p* = 0.03. # ANOVA (LSD post hoc): I vs. II *p* = 0.013; II vs. III *p* = 0.052. † Variable applicable only to hemodialysis groups (I and III). ‡ Comparison only between hemodialysis groups (I vs. III) for † variables. Data are presented as mean ± SD unless otherwise specified.

**Table 3:** Glycemic parameters (PG_3_m, plasma glucose, HbA1c and GA) across the three study groups.

Variable	I Group HD+DM+ *n* = 40	II Group HD–DM+ *n* = 15	III Group HD+DM– *n* = 22	*p*-Value
PG_3_m (mmol/L) †	8.2 ± 2.4	9.0 ± 1.5	4.9 ± 0.7	<0.001 *
Plasma glucose (mmol/L) †	8.4 ± 4.3	8.5 ± 2.8	5.3 ± 1.0	0.030 **
GA (%)	11.3 ± 1.8	9.8 ± 1.3	6.6 ± 1.3	<0.001 #
HbA1c (%)	6.7 ± 1.3	8.7 ± 1.6	5.1 ± 0.6	<0.001 ***

† Fasting measurements. PG_3_m represents the arithmetic mean of three-monthly fasting PG values. * ANOVA (LSD): I vs. III *p* < 0.001; II vs. III *p* < 0.001. ** ANOVA (LSD): I vs. II *p* = 0.001; II vs. III *p* = 0.008. *** ANOVA (LSD): I vs. II *p* < 0.001; I vs. III *p* < 0.001; II vs. III *p* < 0.001. # ANOVA (LSD): I vs. II *p* = 0.007; I vs. III *p* < 0.001; II vs. III *p* < 0.001. Values expressed as mean ± SD. ANOVA overall *p*-values shown in table.

**Table 4:** Correlation analysis between PG_3_m, GA and HbA1c in diabetic groups.

Variable	Group	ρ	*p*-Value
PG_3_m vs. HbA1c	HD+DM+	0.768	<0.001
PG_3_m vs. GA	HD+DM+	0.599	<0.001
HbA1c vs. GA	HD+DM+	0.680	<0.001
PG_3_m vs. HbA1c	HD–DM+	0.670	0.006
PG_3_m vs. GA	HD–DM+	0.323	0.240
HbA1c vs. GA	HD–DM+	0.603	0.017

ρ = Spearman’s rank correlation coefficient. *p* values < 0.05 were considered statistically significant. Abbreviations: GA, glycated albumin; HbA1c, glycated hemoglobin; PG_3_m, 3-month mean plasma glucose.

**Table 5:** Logistic Regression Analysis: (HD+DM+ Group).

Variable	Univariate OR	95% CI	*p*-Value	Multivariate OR	95% CI	*p*-Value
PG (mmol/L)	1.63	1.147–2.317	0.006	—	—	—
PG_3_m (mmol/L)	6.60	2.283–19.081	<0.001	3.96	1.061–14.780	0.041
HbA1c (%)	7.09	2.361–21.315	<0.001	—	—	—
GA (%)	3.30	1.902–5.722	<0.001	3.23	1.543–6.765	0.002

ORs reflect the change in the odds of poor glycemic control per 1-unit increase in each biomarker. PG was excluded from the multivariate analysis due to collinearity with PG_3_m, while HbA1c was not entered into the multivariate model because of collinearity with both PG_3_m and GA. The multivariate model was adjusted for serum hemoglobin and albumin as pre-specified covariates. Variables with *p* < 0.10 in univariate analysis were eligible for inclusion. OR, odds ratio; CI, confidence interval.

**Table 6:** Clinical and laboratory parameters according to GA and HbA1c categories.

Variable	GA < 10% (*n* = 9)	GA > 10% (*n* = 31)	*p*-Value (GA Groups)	HbA1c < 6.5% ((*n* = 20)	HbA1c > 6.5% (*n* = 20)	*p*-Value (HbA1c Groups)
Age (years)	61.67 ± 14.42	59.65 ± 12.77	0.68	61.65 ± 10.77	58.60 ± 15.04	0.55
Waist circumference (cm)	95.33 ± 18.24	91.71 ± 14.12	0.53	94.55 ± 16.23	90.50 ± 4.57	0.39
BMI (kg/m^2^)	24.80 ± 5.85	23.69 ± 5.06	0.56	25.26 ± 5.55	22.61 ± 4.57	0.11
Hemoglobin (g/dL)	10.98 ± 1.08	10.56 ± 1.34	0.57	10.88 ± 0.96	10.12 ± 1.46	0.06
ESA dose (IU/week)	6300 [3000–9500]	6200 [2500–10,500]	0.98	5400 [2000–8000]	7200 [3000–11,000]	0.34
Kt/V	1.32 ± 0.34	1.24 ± 0.32	0.65	1.32 ± 0.35	1.19 ± 0.30	0.22
CRP (mg/L)	12.0 [4.2–18.5]	22.0 [6.5–40.0]	0.12	5.1 [3.0–7.5]	20.0 [6.0–33.0]	0.11
Serum albumin (g/L)	35.03 ± 7.03	36.44 ± 1.13	0.55	36.50 ± 3.16	34.35 ± 8.20	0.31

Note: Variables with normal distribution are presented as Mean ± SD. Variables with non-normal distribution (ESA dose, CRP) are presented as Median [IQR]. Comparisons performed using independent samples *t*-test (normal distribution) or Mann–Whitney U test (non-normal distributions).

## Data Availability

The data presented in this study are available on request from the corresponding author. The data are not publicly available due to patient privacy and institutional restrictions.

## Abbreviations

AUC: area under the curve; CI, confidence interval; CKD, chronic kidney disease; CRP, C-reactive protein; CV, cardiovascular; DM, diabetes mellitus; ESA, erythropoiesis-stimulating agent; GA, glycated albumin; HbA1c, glycated hemoglobin; HD, hemodialysis; HR, hazard ratio; IQR, interquartile range; Kt/V, dialysis adequacy index; OR, odds ratio; PG, plasma glucose; PG_3_m, 3-month mean plasma glucose; ROC, receiver operating characteristic; SD, standard deviation; T2DM, type 2 diabetes mellitus.
